# Contribution of Individual-based Models in malaria elimination strategy design

**DOI:** 10.1186/1475-2875-9-S2-P9

**Published:** 2010-10-20

**Authors:** Jordi Ferrer, Clara Prats, Daniel López, Joaquim Valls, Domingo Gargallo

**Affiliations:** 1 Departament de Física i Enginyeria Nuclear, Escola Superior d'Agricultura de Barcelona, Universitat Politècnica de Catalunya, Castelldefels (Barcelona), Spain; 2 Grupo Ferrer, Barcelona, Spain

## Background

Global strategies to fight malaria consist of three components: medical coverage scale-up in the affected regions, sustained control of the disease and increasing local elimination. These strategies normally consider long-term temporal scales of the order of the decade and are typically formulated either in technical terms or through mathematical models that are not easily communicated to non-experts (e.g. local people that act as malaria control technicians and local governments).

Yet, global strategies finally lie on local specific interventions, carried out by agents with a limited scope of action and covering short spans. Field actions against malaria typically have to struggle against logistic limitations and must be very well coordinated in order to succeed. There is a need for models that can connect field actors with strategy designers in order to tackle the specific constraints of each particular intervention, and to redefine objectives on the fly, in accordance with the field results.

## Methods

The present work proposes a formalism to plan, communicate and discuss specific interventions with Individual-based models (IbMs). In that way, an IbM has been built and implemented in a user-friendly free simulating platform called NetLogo. Three representative epidemic models are presented and compared with each other: a simple SIR epidemic model [1], a continuous model that considers host and vector populations and the IbM.

## Results

The three models provide equivalent results for an example case study. Nevertheless, each of them is more suitable in a specific context of application and use. IbM is the best choice to plan and analyse actions at the local level, and its implementation in a user-friendly platform such as NetLogo facilitates its communication to non-experts. Moreover, all three models agree in the strategies that lead to elimination of the disease through including actions at different levels and targeting clinically asymptomatic ill people, which definitively are the long-lasting pool of the parasite.

## Conclusions

Malaria control and elimination requires the coordination of multiple actors and the strong commitment of the local community which may be affected by logistic and communication limitations. This makes the scheduling and assessment of the ongoing strategies a central issue in the fight against malaria. IbM is a valuable tool to plan, analyse and communicate interventions.
